# Rethinking the nature of medicine: limits of the inquiry thesis through the case of African traditional medicine

**DOI:** 10.1007/s11017-025-09732-3

**Published:** 2025-10-02

**Authors:** Likhwa Ncube

**Affiliations:** Centre for Philosophy of Epidemiology, Medicine, and Public Health (CPEMPH), Faculty of Humanities, University of Johannesburg, Auckland Park, PO Box 524, Johannesburg, 2006 South Africa

**Keywords:** Nature of medicine, Curative thesis, Inquiry thesis, African traditional medicine, Refined curative thesis, Prediction and understanding

## Abstract

This paper critically evaluates Alex Broadbent’s Inquiry Thesis, which defines medicine by the core competencies of prediction and understanding. While the thesis seeks to avoid parochialism by identifying features that could apply across traditions, its applicability falters when examined against African traditional medicine (ATM). Using ATM as a test case, this paper demonstrates that legitimate medical systems can operate coherently and enjoy broad social legitimacy without consistently exercising prediction or understanding in Broadbent’s sense. This challenges the Inquiry Thesis both as a general account of medicine and as an inclusive framework. Accordingly, this paper tentatively advances a Refined Curative Thesis (RCT), which shifts the unifying criterion from competencies to aims: medicine is defined as the organized, socially recognized effort to restore or maintain health through interventions directed at beneficial change. RCT accommodates epistemic diversity, preserves conceptual discipline, and avoids collapsing into unstructured pluralism. By centring the curative aim, this revised account offers a more inclusive, philosophically robust, and culturally sensitive definition of medicine. The analysis deepens philosophical understanding of medicine while also bearing practical significance for global health policy, intercultural dialogue, and the recognition of diverse medical systems.

## Introduction

Alex Broadbent has recently reignited philosophical debate by asking, somewhat provocatively, why so little attention has been paid to the foundational question of *what medicine is* [1–3]. While much has been written about medical ethics, epistemology, and methodology, Broadbent contends that the very nature of medicine itself has been comparatively neglected. I largely agree with this observation. His claims have sparked significant discussion [4–9], drawing attention to questions about medicine’s aims, its epistemic capacities, and its nature.

Broadbent positions himself against the traditional and what might fairly appear intuitive, the “Curative Thesis,” which holds that the ability to cure is an essential defining feature  of the nature of medicine. Instead, he advances the “Inquiry Thesis,” according to which medicine’s defining features are its competencies of prediction and understanding [1, 2]. This move is meant to make the definition less parochial, avoiding a model that privileges only Western biomedicine by identifying competencies that could, in principle and practice, be shared across medical traditions [2].

Yet, despite Broadbent’s stated goal of inclusivity, the Inquiry Thesis he proffers is framed around competencies most clearly exemplified in Western biomedicine. Ironically, these competencies are not *reliably* identifiable in certain non-Western traditions, most notably African traditional medicine (ATM), which means the framework risks excluding precisely the diversity of traditions it was meant to accommodate.

This paper examines this tension through a sustained case study of ATM. The choice of ATM is not incidental: it remains a major medical system for millions of people in sub-Saharan Africa. Particularly in Southern Africa, surveys suggest that up to 80% of the Black South African population consults traditional healers [10, 11]. ATM is not merely a set of spiritual or cultural practices; it is recognized under national health regulations in South Africa and elsewhere as a legitimate part of the health sector [12]. For this reason, ATM offers a valuable test case for evaluating whether Broadbent’s account can truly encompass global medical diversity.

The aim of this paper is not to reject the Inquiry Thesis outright for parochialism, but to examine its underlying assumptions and to test its limits. By scrutinizing ATM, I argue that ATM provides a decisive counterexample to the Inquiry Thesis. I show that the competencies of prediction and understanding do not consistently function in this tradition as Broadbent defines them. This exposes a double failure of the Inquiry Thesis:

1. As a general account, it identifies competencies that are not universal to all medical traditions.
2. As an inclusive framework, it risks marginalizing precisely those non-Western traditions it seeks to accommodate.

The paper’s purpose is primarily critical. However, critique without any constructive orientation risks leaving the definitional project incomplete. While a complete replacement for the Inquiry Thesis lies beyond the scope of this paper, I offer a tentative alternative in the form of a *Refined Curative Thesis (RCT)*. RCT re-centers the curative aim, understood broadly as the organized, socially recognized effort to restore or maintain health through interventions directed at beneficial change. This retains conceptual discipline while avoiding the parochialism of epistemic gatekeeping. Prediction and understanding remain important as instruments, but they are not universal criteria for inclusion.

The paper proceeds as follows. The second section analyses Broadbent’s critique of the Curative Thesis and his defence of the Inquiry Thesis. The third section outlines ATM as a medical tradition, focusing on its epistemic commitments, practices, and social roles. The fourth section evaluates the Inquiry Thesis in light of ATM, showing where prediction and understanding fail to serve as universal competencies. The fifth section develops the double failure of the Inquiry Thesis and sketches the Refined Curative Thesis as a more inclusive alternative. And the sixth section concludes by highlighting the implications of this shift for philosophical debates on the nature of medicine and for global health policy.

## Broadbent on the nature of medicine

Broadbent’s recent work has revitalized discussion on the fundamental question of the nature of medicine by challenging the intuitive assumption that medicine is primarily defined by its ability to cure disease. Broadbent [2, 3] notes that medicine across history has often lacked reliable cures, yet has persisted as a respected professional practice. He reasons that if curing were truly the *core* of medicine, traditions with limited curative success should have disappeared. The historical endurance of medicine, despite frequent curative failure, demands a different explanation.

Broadbent advances two major theses in this context: the *Curative Thesis* and the *Inquiry Thesis*. The Curative Thesis states that medicine’s defining aim is “the sustained and organized effort to heal the sick, or prevent them from getting sick in the first place” [2, p. 35]. Broadbent adopts a generous definition of “cure” to include not only complete eradication of disease but also interventions that alleviate symptoms, reduce suffering, or extend life [1]. This framing allows palliative and preventive measures to count as curative in a broad sense.

However, Broadbent ultimately rejects the Curative Thesis. He argues that the historical and cross-cultural record undermines its emphasis on cure and treatment as the defining feature of medicine: medicine has frequently failed to deliver cures, especially prior to the twentieth century [13, 14]. The analogy he offers is striking; professions such as blacksmithing or taxi-driving cannot survive long without producing functional tools or transporting passengers. By contrast, medicine has continued despite long stretches of poor curative success. Broadbent’s answer to this puzzle is that medicine persists because it provides something more fundamental than cures: the competencies of *prediction* and *understanding*.

This leads to the Inquiry Thesis, which defines medicine not by its curative capacity but by its *epistemic competencies*. Broadbent distinguishes between two key capacities:

1. *Understanding Thesis*: “The ability to engage meaningfully with the project of attaining understanding of health and illness is essential to medicine.”
2. *Predictive Thesis*: “The ability to make good predictions about health states, including conditional predictions, is essential to medicine” [2, p. 65].

Broadbent uses examples to illustrate these claims. Consider the case of a patient with a necrotic finger [2]. The physician predicts accurately that the finger will change colour and fall off, even though no cure is offered. This is still recognized as legitimate medical practice because the doctor demonstrates both understanding of the pathology and predictive competence regarding its progression.

Prediction, in Broadbent’s account, is not merely an optional skill but a logical precursor to cure. For an intervention to qualify as a genuine cure (rather than a fortunate accident – luck), recovery must be predicted on the basis of the treatment, and a worse outcome must be predicted without it. He gives the counterexample of a burglar who recovers from illness after randomly ingesting pharmacy drugs; this, for Broadbent, cannot count as a medical cure because it lacks both predictive basis and explanatory grounding [2].

Understanding is equally central. Historically, periods of major therapeutic breakthroughs have followed advances in medical understanding, particularly after the scientific revolution and into the twentieth century [15]. For Broadbent, this reinforces that prediction and understanding are *primary competencies*, while cure is *secondary*, emerging from their successful application.

In defending the Inquiry Thesis, Broadbent explicitly positions it as a cross-cultural account. He acknowledges that medical traditions vary widely but insists that prediction and understanding are the shared competencies that persist across time and place [2]. This move is intended to avoid privileging Western biomedicine. However, as later sections will show, his selection of competencies may itself be culturally loaded. While prediction and understanding are robustly present in Western medicine, their role in traditions such as ATM is more complex.

Broadbent’s account has not gone unchallenged. Critics such as Metz [6] and Harris [5] have questioned both the descriptive adequacy and conceptual coherence of the Inquiry Thesis. Metz points to medicine’s long history of false explanatory frameworks (e.g., humoral theory, miasma theory) to question whether ‘understanding’ should be considered a core competency when so much of it has been wrong. Harris, examining patient movement between ATM and biomedicine, observes that patients’ choices are often driven less by explanatory capacity than by pragmatic hope for a cure—casting doubt on whether understanding is primary in practice.

These objections highlight the importance of examining Broadbent’s Inquiry Thesis against concrete traditions beyond Western medicine. ATM provides a particularly instructive case: it is a long-standing medical system that enjoys broad social legitimacy, yet its epistemological foundations and practical methods do not consistently match Broadbent’s prediction-and-understanding framework. In the following section, I outline ATM in detail to provide the necessary context for testing the Inquiry Thesis against this alternative tradition.

## African traditional medicine

African traditional medicine (ATM) encompasses a diverse set of indigenous medical practices, belief systems, and knowledge traditions developed and transmitted across generations throughout the African continent [16–18]. Although ATM varies by region and culture, it is unified by its holistic approach to health, integrating physical, spiritual, and social dimensions of well-being. It is neither a peripheral nor declining tradition: in South Africa, for example, up to 80% of Black South Africans consult traditional healers at some point [10]. ATM is also institutionally recognized [11]. The South African Traditional Health Practitioners Act (Act No. 22 of 2007) formally regulates traditional healers, underscoring that ATM is part of the national health framework rather than merely an informal spiritual practice [12].

### Holistic conception of health

ATM conceptualizes health as the harmonious balance of bodily, spiritual, and social elements. Illness is often interpreted as the disruption of this equilibrium, sometimes through biological causes, but frequently through social disharmony, ancestral displeasure, or malevolent spiritual forces [16, 19]. This epistemological orientation contrasts sharply with the disease ontology of Western biomedicine, which *largely* localizes illness in physical pathology [20]. In ATM, restoring health typically involves simultaneous attention to the physical body, spiritual relations, and social environment.

### Role of healers and spirituality

Traditional healers, variously known in Southern Africa as *sangomas*, *inyangas*, diviners, or herbalists, are central to ATM’s operation. They serve not only as therapeutic practitioners but also as custodians of social and spiritual order. Their expertise is grounded in an apprenticeship process that typically begins with dreams or visions interpreted as a “calling” from ancestors, leading to initiation (*ukuthwasa*) [16, 21]. The healer’s diagnostic and therapeutic actions are thus epistemologically embedded in ancestral communication.

Spirituality is not an optional dimension of ATM practice but constitutive of its methodology. For example, in Southern Africa, the spirit of a deceased relative (*idlozi*) may be understood to inhabit the healer temporarily, offering guidance for diagnosis and treatment [22]. This model departs from the biomedical ideal of explanation grounded in mechanistic understanding, but it is nonetheless authoritative within the cultural epistemology of ATM.

### Herbal medicine and indigenous knowledge

An important domain of ATM is herbal medicine. Indigenous pharmacopeia, transmitted orally across generations, encompasses a wide range of plant-based remedies for ailments ranging from common colds to chronic illnesses [17, 21]. While some herbal formulations have been investigated scientifically, leading to recognition of their pharmacological properties, many remain understudied, partly due to epistemic biases in global health research [19]. The persistence of herbal remedies in ATM reflects both their perceived efficacy and the epistemic authority of the healer’s expertise in preparing, dosing, and contextualizing their use.

### Social and communal dimensions of healing

ATM operates within a fundamentally communal framework. Illness is often conceptualized as not solely affecting an individual but as embedded in the family or community network [17]. In some cases, such as those involving spiritual attacks, the condition is thought to implicate entire kinship groups, necessitating collective consultation and ritual participation [16, 23]. Healing rituals, such as community cleansing ceremonies, serve both therapeutic and social-cohesive functions, reaffirming community bonds while addressing perceived causes of illness.

### ATM as a medical tradition

One important question for philosophical analysis is whether ATM should be classified as a medical tradition or as a form of spiritual or religious practice. This is especially pressing in the context of Broadbent’s Inquiry Thesis, which is intended to cover ‘medicine’ across traditions. While ATM includes elements that might resemble religious ritual to external observers, it is institutionally and socially understood as a form of healthcare amongst the greater African people [18]. Of course, healthcare—and healing more broadly—is understood within ATM as an interconnected relationship between the biological, social, and spiritual. National health policies in multiple African states, including South Africa, Ghana, and Mali, officially incorporate ATM into their health systems [12, 18]. Patients seek out ATM not primarily for abstract spiritual benefits but for concrete interventions aimed at alleviating illness, restoring health, and preventing future disease [5]. This practical orientation justifies its consideration as a medical system within comparative philosophy of medicine.

Thus, ATM represents a long-standing, culturally embedded, and socially recognized form of medical practice. Its epistemic foundations, which integrate spiritual revelation, community involvement, and herbal pharmacology, present an important challenge for any universal account of the nature of medicine; ATM’s conception of healing is biological, social, and spiritual. The following section will test whether Broadbent’s core competencies – prediction and understanding – are meaningfully and consistently present in ATM, and thus whether the Inquiry Thesis can successfully encompass this tradition.

## Inquiry thesis and African traditional medicine

The preceding section presented ATM as a socially recognized, institutionally regulated, and widely practiced medical tradition. The present section tests Broadbent’s Inquiry Thesis against ATM in detail. Broadbent identifies two core competencies as defining the nature of medicine: prediction and understanding. These competencies are intended to be universal across time, tradition, and place [2]. The key question, then, is whether they are reliably present in ATM in the form that Broadbent envisions.

In this section, I argue that these competencies are not consistently exercised in ATM as Broadbent defines them, and that the way competence is recognized in ATM diverges from his framework. This is not merely a descriptive difference; it has direct implications for the Inquiry Thesis, because Broadbent’s account requires these competencies to be *reliably* identifiable across all traditions in order to function as the unifying definition of medicine [2, 3].

### Broadbent’s argument from inference to the best explanation

Broadbent’s first major defence of the Inquiry Thesis rests on *inference to the best explanation* [2, 24]. His reasoning is straightforward: if a patient regards a practitioner as competent despite the absence of a cure, the best explanation is that the practitioner has demonstrated medical expertise through understanding and prediction. For example, Broadbent’s necrotic finger case involves a physician who predicts the progression of the condition (color change, eventual detachment) without offering a cure. The physician is nonetheless recognized as competent because they have demonstrated both understanding of the pathology and predictive skill.

Applying this to ATM raises a significant challenge. In ATM, when a patient’s illness persists despite treatment, the best explanation the patient offers for the persistence of illness rarely has anything to do with prediction or understanding as Broadbent conceives them. Instead, patients’ inferences to the best explanation typically attribute the lack of improvement to:

- The superior spiritual power of an adversary [16, 17].
- Ancestral displeasure or unmet ritual obligations [21, 23].
- The healer’s misalignment with certain spiritual forces or the need for a more powerful healer [19, 22, 23].

In such cases, the healer is still regarded as competent not because they predicted the course of the illness or explained its causal mechanism in biomedical terms, but because they remain engaged in the process of *cure-seeking*. The healer’s ongoing competence is rooted in persistence, ritual skill, and alignment with spiritual epistemologies.

This difference is more than cultural rendition; it is philosophically crucial. Broadbent’s account assumes that recognition of competence is tied to predictive and explanatory performance, ATM shows that recognition of competence can be grounded in other epistemic and practical commitments. For the Inquiry Thesis to hold universally, it would need to explain how these forms of competence are also reducible to prediction and understanding, or else accept that the framework does not cover ATM.

### Prediction in ATM: conditionality, reliability, and context

Broadbent’s second defence of the Inquiry Thesis stresses the logical primacy of prediction over cure. For a cure to count as a genuine expression of medical competence, recovery must be predicted with treatment and deterioration predicted without it. This, for Broadbent, is the key to avoiding the attribution of “cure” to luck [2]. In ATM, however, predictive claims are rarely expressed in the conditional form Broadbent envisions. In trance-healing and other diagnostic practices, healers typically do not frame outcomes in terms of explicit counterfactual reasoning: *If you follow this intervention, recovery will occur; if you do not, the condition will worsen.* Instead, predictions are often embedded in a spiritual narrative. A *sangoma* might say that an illness will improve if the patient performs certain rituals or makes offerings to appease the ancestors [22, 23].

While such statements have a predictive element, they are not structured as testable hypotheses in both the biomedical and Broadbent’s senses. They are conditional only within the logic of the spiritual framework: recovery is contingent on spiritual alignment, not on physiological causation as Western medicine understands it [16, 19].

Moreover, the *reliability* of predictions in ATM is evaluated differently. In biomedicine, prediction is often validated through repeated testing, population-level data, and mechanisms of falsification. In ATM, predictions (if present) are validated through the congruence of the healer’s statements with cultural expectations, the perceived sincerity of the ritual process, and the eventual improvement (which may be attributed to factors beyond the immediate treatment). This is not to say ATM lacks predictive capacity; rather, it exercises prediction according to its own epistemic norms. The challenge for Broadbent is that his Inquiry Thesis requires prediction to be a shared, *reliably* identifiable competence across all traditions. In ATM, prediction is often implicit, non-standardized, and expressed in non-counterfactual terms.

### Understanding in ATM: the trance-healing challenge

Understanding presents an even sharper challenge to the Inquiry Thesis. Broadbent’s ‘understanding thesis’ requires that medicine engage meaningfully in attaining understanding of health and illness. Understanding is framed as explanatory, with conceptual grasp of causal mechanisms playing a central role [2]. In ATM, particularly in trance-healing, the healer’s role is not to demonstrate mechanistic comprehension but to act as a conduit for knowledge from spiritual agents. As Mncube [22] describes:

Briefly, here is how a *sangoma* takes themself to diagnose illness through the methods of trance-channeling and divination bones, respectively. In the first instance, a client will consult with a *sangoma* as a result of the client’s self-assessed psychosocial, physiological, or spiritual illness. The diagnosis and explanation of illness is then clarified by the *sangoma*. If the *sangoma* uses trance-channeling as a diagnostic method, then in the consultation the *sangoma* will take themselves to be occupied or possessed by their own ancestors. In this trance-like state, it is believed that the *sangoma*’s ancestors communicate through the *sangoma* as a medium to the client, to deliver personalized diagnostic information and guidance about the client’s illness. [22, p. 233]

It is crucial to notice as per the quotation above that in trance-channeling, the *sangoma* is occupied or possessed by their ancestors, who speak through them to deliver diagnostic information and guidance. The *sangoma* does not claim independent comprehension of the illness; instead, they relay the knowledge imparted by the ancestral spirit. This distinction is philosophically significant. In Broadbent’s terms, ‘understanding’ is cognitive possession of explanatory relations. It is predicated on there being one (someone) who understands behind the understanding. In trance-healing, the authority rests on accurate transmission of ancestral instruction, not personal explanatory knowledge. The healer’s competence is measured by their ritual ability/capacity to receive, interpret, and enact spiritual knowledge, not by their explanatory account of disease.

This is strikingly similar to Metz’s “oracle” thought experiment [6], in which an oracle can direct effective healing without possessing scientific understanding. If the oracle case counts as medicine without mechanistic understanding, Broadbent’s framework would need to stretch “understanding” to include ‘non-explanationist’ epistemologies [9]. But this would blur the very conceptual boundaries that give the Inquiry Thesis its normative force.

### Patient explanations and the recognition of competence

Why does it matter how patients explain competence? The Inquiry Thesis uses patient recognition of competence as part of its justification for prediction and understanding as universal features. Broadbent argues that even when cures fail, patients continue to trust practitioners who display these competencies [2].

In ATM, however, patient trust is sustained through different mechanisms, for one, relational trust. This can be best described as involving ongoing care and attention by the healer, reinforcing the patient’s confidence. Another aspect is spiritual alignment, specifically the healer’s ability to navigate ancestral relationships and spiritual obligations, which validates their role. One could also add community validation here; that is, shared cultural understandings of illness and recovery support continued engagement. These mechanisms do not require prediction or understanding in Broadbent’s terms. This difference matters because it shows that the Inquiry Thesis is built on an empirical assumption that may hold in biomedicine but does not generalize well to ATM.

## Why Broadbent’s framework cannot simply expand to include ATM’s epistemology

Could the Inquiry Thesis be saved by expanding the definitions of prediction and understanding to include ATM’s spiritual epistemology? For example, could one argue that a trance-healer’s receipt of ancestral guidance constitutes ‘understanding’ within that tradition, and that a divination-based diagnosis is a form of ‘prediction’?

This approach risks collapsing the Inquiry Thesis into a thin pluralism. Prediction and understanding in the Inquiry Thesis are meant to be *common, reliable competencies* across traditions. Expanding them to cover any tradition’s internal epistemology makes them so broad as to lose discriminatory power. At that point, the Inquiry Thesis ceases to function as a precise philosophical definition and becomes a culturally relative description. Broadbent’s own aim is to avoid precisely this move, seeking a cross-cultural core that avoids parochialism without losing definitional rigor. ATM shows that his chosen competencies cannot serve this role without either excluding legitimate traditions or diluting the criteria beyond usefulness.

ATM presents a sustained challenge to the Inquiry Thesis. Prediction and understanding, as Broadbent defines them, are not *reliably* identifiable in ATM. Competence in ATM is recognized through different epistemic norms – persistence in cure-seeking, spiritual authority, and relational trust, for example, rather than predictive and explanatory success. This is not a failure of ATM; it is a limitation of the Inquiry Thesis. If the Inquiry Thesis cannot accommodate ATM without distorting either the tradition or the thesis itself, then its claim to universality is undermined.

The next section will explore the implications of this conclusion. Suppose I am correct that prediction and understanding are not universal competencies of medicine. In that case, one must either revise the Inquiry Thesis or replace it with an alternative that can accommodate traditions like ATM without losing conceptual clarity.

## Inquiry thesis fails doubly

The preceding section examined ATM through the lens of the Inquiry Thesis, focusing on Broadbent’s two core competencies: prediction and understanding. The conclusion was that these competencies, as formulated by Broadbent, do not *reliably* characterize ATM. In this section, I draw out the philosophical implications of this finding. Broadbent’s framework fails doubly: first, it fails as an account of the nature of medicine in general; second, it fails to fulfil its own ambition of encompassing non-Western medical traditions such as ATM.

At this juncture, there are three possible responses to this failure:

1. Reject or modify the Inquiry Thesis (to include competencies beyond prediction and understanding).
2. Deny that ATM is a legitimate medical tradition (thereby preserving the Inquiry Thesis as formulated).
3. Propose an alternative account of medicine (one that better accommodates ATM while maintaining a coherent definition).

I will address each in turn, beginning with pluralism and the Inquiry Thesis itself, before moving to ATM’s legitimacy and lastly, a Refined Curative Thesis as an alternative framework.

### The pluralism question: can one abandon a single defining core?

Broadbent explicitly rejects a pluralistic approach to defining medicine:

I acknowledge that the core medical competence might differ in different times and places, and that my quest may therefore be hopeless… But in my view, it is always worth starting out hopeful. If we assume that the various medical traditions in different times and places have something in common that identifies them as medical, and then set about finding it, we may find something that we would not find if we started out pluralists. Implausible assumptions are sometimes the most productive. [2, p. 62]

Broadbent’s rejection of pluralism is based on the idea that pluralism risks collapsing into vagueness, leaving one without a clear, principled account of what unites different medical traditions. His concern mirrors debates in philosophy of science about “cluster concepts” such as  game, art, or health. These concepts accommodate diversity by listing overlapping characteristics rather than a single necessary and sufficient condition [25].

In the case of medicine, a pluralist might argue that there is no single universal competency (prediction, understanding, cure, or otherwise) that appears in every medical tradition. Instead, there is a *family resemblance* among different practices: some traditions emphasize herbal pharmacology, some rely on spiritual diagnosis, others stress empirical observation, but all occupy a shared cultural category of “medicine” due to overlapping aims, roles, and methods [6]. While this approach avoids forcing a single competency across diverse traditions, it risks becoming analytically inert. If any culturally recognized healing practice can count as medicine without a clear criterion, then the term ‘medicine’ loses the explanatory precision Broadbent is seeking. The pluralist position might succeed descriptively but fail normatively, leaving one without a principled way to distinguish legitimate medicine from, for example, pseudo-medicine or purely religious rituals.

My rejection of pluralism aligns with Broadbent on this point: medicine needs a definition that is more than a sociological/anthropological list of practices. However, unlike Broadbent, I am open to rethinking what the unifying element might be. The Inquiry Thesis identifies prediction and understanding; I argue that these are not sufficient. But this does not mean abandoning the search for a unifying feature. Instead, I propose refining the Curative Thesis to play this role (see the ‘Towards a refined curative thesis’ section below).

### ATM’s legitimacy as medicine

Another possible way to preserve the Inquiry Thesis without modification would be to argue that ATM is not, in fact, a legitimate medical tradition. This position would hold that ATM’s failure to demonstrate prediction and understanding (in Broadbent’s sense) disqualifies it as ‘medicine.’ This view, however, is untenable for both descriptive and normative reasons:

**Descriptively**: ATM is recognized as part of the health sector in multiple African countries. In South Africa, the Traditional Health Practitioners Act establishes legal categories for different types of healers (herbalists, diviners, traditional birth attendants) and regulates their practice [12]. The World Health Organization similarly includes traditional medicine in its strategic framework for health systems strengthening [26]. These institutional recognitions are not mere cultural niceties; they have material effects on healthcare policy, resource allocation, and patient care.

**Normatively**: Denying ATM’s status as medicine risks a parochialism that Broadbent himself seeks to avoid. If a definition of medicine excludes traditions that millions of people recognize as their primary source of healthcare, then that definition is both descriptively inaccurate and ethically problematic [27]. It would marginalize large populations and undermine cross-cultural dialogue about healthcare policy.

The better philosophical move is to accept ATM as medicine and adapt one’s theory accordingly. One might attempt to do this by, for instance, broadening the definitions of ‘prediction’ and ‘understanding’ to include ATM’s spiritual epistemology. For example, a trance-healing session could be reframed as a form of ‘understanding’ in which the healer grasps the causal structure of illness in spiritual rather than mechanistic terms. However, even for this move, too, I argued above that it eventually undermines the precision of the Inquiry Thesis.

That is, Broadbent’s competencies are meant to be generalizable and assessable across traditions. If ‘understanding’ is expanded to mean any culturally internal account of causation, whether or not it is explanatory in a scientific sense, then the Inquiry Thesis collapses into a form of pluralism it was designed to avoid. Similarly, if ‘prediction’ includes any pronouncement about outcomes, regardless of its conditional structure or testability, the competency becomes so elastic that it loses normative force.

### The double failure of the inquiry thesis

I am now in a position to state the double failure more explicitly. First, failure as a general account of medicine. By privileging prediction and understanding, the Inquiry Thesis fails to identify competencies that are present in all medical traditions. ATM demonstrates that legitimate medical traditions can function with minimal or no exercise of these competencies as Broadbent defines them. Second, failure as an inclusive framework. Broadbent’s project is motivated by the desire to avoid Western parochialism, yet his chosen competencies are culturally specific. By setting prediction and understanding as the standard, the Inquiry Thesis risks excluding traditions like ATM despite their recognized medical status.

The Inquiry Thesis thus fails doubly, not because its ambition is misguided, but because its execution is too narrow. A broader, goal-oriented conception of medicine, similar to the one I articulate below, is necessary to capture its nature across cultures and historical contexts.

### Towards a refined curative thesis

Although the central aim of this paper has been to evaluate the adequacy of the Inquiry Thesis when applied to ATM, the analysis necessarily invites consideration of what might replace it. A constructive proposal is not the primary focus of this paper; however, a tentative alternative is nonetheless critical, as critique without a viable replacement leaves the theoretical landscape incomplete. In that spirit, I offer here a preliminary sketch of what I will call the *Refined Curative Thesis* (RCT).

The RCT I propose retains the curative aim at the center of medicine’s identity, but in a broader, more flexible sense. For RCT: ‘Medicine is the organized, socially recognized practice directed towards the restoration, preservation, or promotion of health through interventions aimed at beneficial change in a patient’s condition, even when such interventions do not reliably result in complete cure’.

This refined version differs from the ‘naïve’ Curative Thesis in several key respects. First, it does not require consistent success at curing disease. Instead, it recognizes that medicine can legitimately persist even when cures are rare or partial, so long as the *aim* remains directed toward health improvement [8]. Second, it accommodates practices where cure is conceptualized differently, as in ATM, where healing may involve restoring harmony between physical, spiritual, and social dimensions rather than eradicating a pathogen [16, 21]. Third, it allows for multiple epistemic routes to curative aims. Whereas the Inquiry Thesis limits legitimacy to systems with prediction and understanding as Broadbent defines them, RCT recognizes that these competencies are valuable instruments but not universal criteria.

Importantly, this approach still preserves *normative boundaries*. It does not collapse into an ‘anything goes’ pluralism. A practice must still be oriented toward health-improving interventions to count as medicine. Systems that make no organized effort to improve health, or which operate in bad faith, fall outside this definition. This retains the conceptual discipline Broadbent seeks while avoiding cultural parochialism.

While the development of a complete replacement for the Inquiry Thesis lies beyond the central scope of this paper, this tentative RCT is an essential step toward that replacement. It takes seriously Broadbent’s concern about parochialism and the need for a framework that can encompass legitimate traditions like ATM without distortion. On this view, medicine’s identity is anchored in its *teleology*, its directedness towards curing or healing, rather than in the particular competencies it employs. Prediction and understanding become important *instruments* of medicine, but they are not necessary conditions for identifying a practice as medicine.

## Conclusion

Broadbent’s Inquiry Thesis is a bold and valuable attempt to articulate a unifying definition of medicine, one that avoids parochialism and captures the nature of the practice across cultures and historical periods. Its ambition deserves serious engagement. However, as the analysis of African traditional medicine (ATM) in this paper has shown, the thesis fails to meet its own standard of inclusivity.

The Inquiry Thesis fails in two respects. First, as a general account of medicine, it selects competencies—prediction and understanding—that are not present in all legitimate medical traditions. ATM demonstrates that a medical system can operate coherently, with broad social recognition, and without consistently exercising prediction or understanding in the sense Broadbent intends. Second, as an inclusive framework, the Inquiry Thesis risks marginalizing precisely those non-Western traditions it aims to accommodate, by making them fit epistemic categories derived primarily from Western biomedicine.

The philosophical work of this paper has been to show that rejecting the Inquiry Thesis does not entail falling into an unconstrained pluralism. Broadbent is right to resist definitions of medicine that collapse into mere lists of disparate practices. What is needed is not a surrender of conceptual discipline but a reorientation of the unifying criterion. The *Refined Curative Thesis* (RCT), proposed here, shifts the focus from competencies to aims. By defining medicine as the organized, socially recognized effort to restore or maintain health through interventions aimed at beneficial change through a broader ‘bio-social-spiritual’ notion of healing, RCT accommodates diverse medical traditions without demanding that they all deploy the same epistemic tools. Prediction and understanding remain important as instruments, but they are no longer gatekeeping criteria.

ATM provides a decisive test case. It is recognized in law and policy as part of the health sector, widely utilized by patients, and centrally oriented toward healing. The fact that its epistemic foundations differ from those of biomedicine does not disqualify it from medicine; it simply demonstrates that a narrow set of competencies cannot exhaustively capture medicine’s nature. By incorporating ATM into the philosophical analysis, one also broadens the discourse beyond Western contexts, responding to the normative concern raised by Broadbent himself that definitions of medicine must avoid cultural bias. At the same time, RCT preserves conceptual coherence, resisting a slide into vague pluralism.

Finally, Broadbent’s Inquiry Thesis, while insightful, ultimately fails as a universal account of the nature of medicine. Its emphasis on prediction and understanding excludes legitimate medical traditions that do not consistently operate in these terms. A revised framework, one that centres the curative aim of medicine, offers a more inclusive, philosophically robust, and culturally sensitive foundation for defining medicine. Such a reorientation not only deepens the philosophical understanding of medicine but also has practical implications for health policy, intercultural dialogue, and the global legitimacy of diverse medical systems. The failure of the Inquiry Thesis is therefore not a setback but an opportunity. It invites a more expansive, yet still disciplined, philosophical project: to articulate an account of the nature of medicine that is both universal in scope and attentive to the epistemic diversity of global medical traditions.
